# Prenatal phenotypes and pregnancy outcomes of fetuses with 16p11.2 microdeletion/microduplication

**DOI:** 10.1186/s12884-024-06702-w

**Published:** 2024-07-22

**Authors:** Fagui Yue, Mengzhe Hao, Dandan Jiang, Ruizhi Liu, Hongguo Zhang

**Affiliations:** 1 Center for Reproductive Medicine and Center for Prenatal Diagnosis, First Hospital, Jilin University, Changchun, 130021 China; 2 Jilin Engineering Research Center for Reproductive Medicine and Genetics, Jilin University, Changchun, 130021 China

**Keywords:** Chromosomal 16p11.2 deletions and duplications, Chromosomal microarray analysis, Prenatal phenotypes, Pregnancy outcomes

## Abstract

**Background:**

Chromosomal 16p11.2 deletions and duplications are genomic disorders which are characterized by neurobehavioral abnormalities, obesity, congenital abnormalities. However, the prenatal phenotypes associated with 16p11.2 copy number variations (CNVs) have not been well characterized. This study aimed to provide an elaborate summary of intrauterine phenotypic features for these genomic disorders.

**Methods:**

Twenty prenatal amniotic fluid samples diagnosed with 16p11.2 microdeletions/microduplications were obtained from pregnant women who opted for invasive prenatal testing. Karyotypic analysis and chromosomal microarray analysis (CMA) were performed in parallel. The pregnancy outcomes and health conditions of all cases after birth were followed up. Meanwhile, we made a pooled analysis of the prenatal phenotypes in the published cases carrying 16p11.2 CNVs.

**Results:**

20 fetuses (20/20,884, 0.10%) with 16p11.2 CNVs were identified: five had 16p11.2 BP2-BP3 deletions, 10 had 16p11.2 BP4-BP5 deletions and five had 16p11.2 BP4-BP5 duplications. Abnormal ultrasound findings were recorded in ten fetuses with 16p11.2 deletions, with various degrees of intrauterine phenotypic features observed. No ultrasound abnormalities were observed in any of the 16p11.2 duplications cases during the pregnancy period. Eleven cases with 16p11.2 deletions terminated their pregnancies. For 16p11.2 duplications, four cases gave birth to healthy neonates except for one case that was lost to follow-up.

**Conclusions:**

Diverse prenatal phenotypes, ranging from normal to abnormal, were observed in cases with 16p11.2 CNVs. For 16p11.2 BP4-BP5 deletions, abnormalities of the vertebral column or ribs and thickened nuchal translucency were the most common structural and non-structural abnormalities, respectively. 16p11.2 BP2-BP3 deletions might be closely associated with fetal growth restriction and single umbilical artery. No characteristic ultrasound findings for 16p11.2 duplications have been observed to date. Given the variable expressivity and incomplete penetrance of 16p11.2 CNVs, long-term follow-up after birth should be conducted for these cases.

## Background

Chromosomal microarray analysis for detecting copy number variations (CNVs) is currently conducted as the first-tier test in prenatal diagnosis and postnatal developmental disorders. With the expanding application of this technique, some pathogenic recurrent CNVs have been successively identified, e.g. 22q11.2, 7q11.23, 17p11.2, and 16p11.2 [1]. For chromosome 16p11.2 locus, five segmental duplications, known as breakpoint (BP)1 to BP5 from telomere to centromere, make this region prone to non-allelic homologous recombination (NAHR), resulting in recurrent deletions and duplications. Typically, two CNVs are observed at this locus: a proximal 593 kb region between BP4 and BP5 (from 29.6 to 30.2 Mb, hg19) and a distal 220 kb region between BP2 and BP3 (from 28.8 to 29.0 Mb, hg19) [2, 3]. As one of the most frequent genomic disorders, 16p11.2 CNVs, commonly referred to as 16p11.2 deletions and duplications, have drawn more and more attention in clinical practice.

According to the OMIM database, 16p11.2 CNVs could be classified into three clinical disorders: 16p11.2 BP4-BP5 deletion (OMIM 611,913), 16p11.2 BP4-BP5 duplication (OMIM 614,671) and 16p11.2 BP2-BP3 deletion (OMIM 613,444). As the most common deleted locus, 16p11.2 BP4-BP5 deletion has been usually associated with a wide range of clinic manifestations, including developmental delay (DD), intellectual disability (ID), autism spectrum disorder (ASD), impaired speech/language, hearing impairment, epilepsy, obesity, vertebral anomalies, macrocephaly and cardiovascular malformation [4–6]. Patients with 16p11.2 BP4-BP5 duplication could exhibit diverse clinic features, such as ID, ASD, attention deficit hyperactivity disorder (ADHD), bipolar disorder (BD), schizophrenia, decreased body mass index (BMI) and reduced head circumference [7–9]. 16p11.2 BP2-BP3 deletion has been associated with early-onset obesity, ID, DD, ASD, schizophrenia, macrocephaly, increased rate of obesity and type 2 diabetes [4, 10]. There are few reports on 16p11.2 BP2-BP3 duplication, and this chromosomal disorder may be implicated in scoliosis [9]. Although the clinic phenotypes of 16p11.2 CNVs are complicated and variable, these characteristics are generally well delineated.

Currently, most studies involving 16p11.2 CNVs are identified through postnatal evaluation. However, the intrauterine phenotypic features associated with 16p11.2 CNVs are not well described, which poses a challenge for genetic counseling and prenatal management for these carriers. To enhance the prenatal knowledge on 16p11.2 CNVs, we present the clinical and molecular findings of 20 cases with 16p11.2 deletions and duplications in the pregnant women who opted for amniocentesis. Additionally, we systematically reviewed the prenatal phenotypes associated with such chromosomal disorders.

## Methods

### Clinical data

This retrospective study was performed from October 2018 to November 2023 and enrolled 20 cases with 16p11.2 microdeletions and microduplications selected from 20,884 pregnant women. These women were referred to the First Hospital of Jilin University for invasive diagnostic testing via amniocentesis. The main indications for prenatal diagnosis included non-invasive prenatal testing (NIPT) for aneuploidy, maternal serum screening results for aneuploidy, ultrasound anomalies (structural or non-structural), parental chromosomal abnormalities, abnormal childbearing history, advanced maternal age, and voluntary request. All pregnancy women accepted routine prenatal ultrasound examinations during the gestation period, and abnormal ultrasound findings were included in the indications for prenatal diagnosis. All couples denied consanguineous marriage, and the pregnant women denied any exposure to teratogenic agents, irradiation, or infectious diseases during this pregnancy in question. After acquiring genetic testing results, all prospective parents received prenatal genetic counselling, and blood samples were collected with informed consent. The study protocol was approved by the Ethics Committee of the First Hospital of Jilin University (No. 2021 − 706), and written informed consent was obtained from all the couples.

### Cytogenetic analysis

Pregnant women underwent amniocentesis for karyotyping analysis with written informed consent. 30 mL of amniotic fluid cells were collected. Routine cytogenetic analysis was performed using G-band metaphases at 400–500 banding resolution, which were prepared from 20 mL of cultured amniotic fluid cells in accordance with standard protocols in our lab. Twenty metaphases were analyzed for all samples according to the International System for Human Cytogenetic Nomenclature 2016.

### Chromosomal microarray analysis (CMA)

The genomic DNA were extracted from the amniotic fluid cells and parental peripheral blood with QIAamp^®^ DNA Blood Mini Kit (Qiagen, Inc., Hilden, Germany) according to the manufacturer’s protocol. Following written informed consents from all pregnancy women, 10 mL uncultured amniotic fluid cells was collected through amniocentesis. Then the procedures are conducted using CytoScan 750 K array (Affymetrix, Santa Clara, CA, USA), in accordance with the manufacturer’s protocol and our previous study [11]. The procedure included genomic DNA extraction, digestion and ligation, PCR amplification, PCR product purification, quantification and fragmentation, labeling, array hybridization, washing and scanning. Thresholds for genome-wide screening were set at ≥ 100 kb for gains and losses. The detected CNVs were comprehensively estimated by comparing them with published literature and the public databases: (1) Database of Genomic Variants (DGV) (DGV, http://dgv.tcag.ca/dgv/app/home), (2) Database of Chromosomal Imbalance and Phenotype in Humans using Ensemble Resources (DECIPHER, http://decipher.sanger.ac.uk/), (3) Clinical Genome Resource (ClinGen, http://www.clinicalgenome.org/), (4) ClinVar (https://www.ncbi.nlm.nih.gov/clinvar/), (5) PubMed (http://pubmed.ncbi.nlm.nih.gov/) and (6) Online Mendelian Inheritance in Man (OMIM, http://www.ncbi.nlm.nih.gov/omim). And all CNVs were classified as pathogenic (P), likely pathogenic (LP), variants of unknown significance (VOUS), likely benign (LB) and benign (B). Genomic positions refer to the Human Genome assembly Dec.2013 (GRCh38/hg38).

### Selection of prenatally detected 16p11.2 microdeletions and microduplications

In order to summarize the prenatal phenotypes of 16p11.2 deletions and duplications in the published reports, we launched a literature review for identifying relevant articles from inception to 2023. Criteria for the selection were defined as English and Chinese languages, 16p11.2 deletions and duplications, CNVs and prenatal phenotypes. The English language database PubMed (https://www.ncbi.nlm.nih.gov/pubmed/) and the Chinese language databases (Wanfang Data and China National Knowledge Infrastructure) were searched. CNVs with chromosome coordinates for all reviewed cases were required to be provided. A string of the following terms and their synonyms was utilized: 16p11.2 deletion/loss, 16p11.2 duplication/gain, prenatal diagnosis, chromosomal microarray analysis, and ultrasound findings/intrauterine phenotype. The combination of subject words and free words was also used for the search. The prenatal phenotypes and pregnancy outcomes for all cases were sorted out in detail. The ultrasound findings were primarily classified into categories such as the skeletal system, cardiovascular system, brain anomalies, renal anomalies, chest anomalies, orofacial region, non-structural anomalies, etc.

### Follow-up outcomes

The follow-up was mainly carried out through telephone interview using the customized questionnaire after all neonates were delivered in our center. The specific follow-up contents included pregnancy outcomes (miscarriages or birth), gestational age at delivery, sex, birth weight/length, ultrasound findings during pregnancy (nervous system, cardiovascular system, craniofacial growth, respiratory system, abdominal abnormalities, urinary system, alimentary system, musculoskeletal system and others), and postnatal health conditions (congenital defects, craniofacial dysmorphisms, skeletal anomalies, developmental details and so on).

## Results

### Study population

**Fig. 1:**
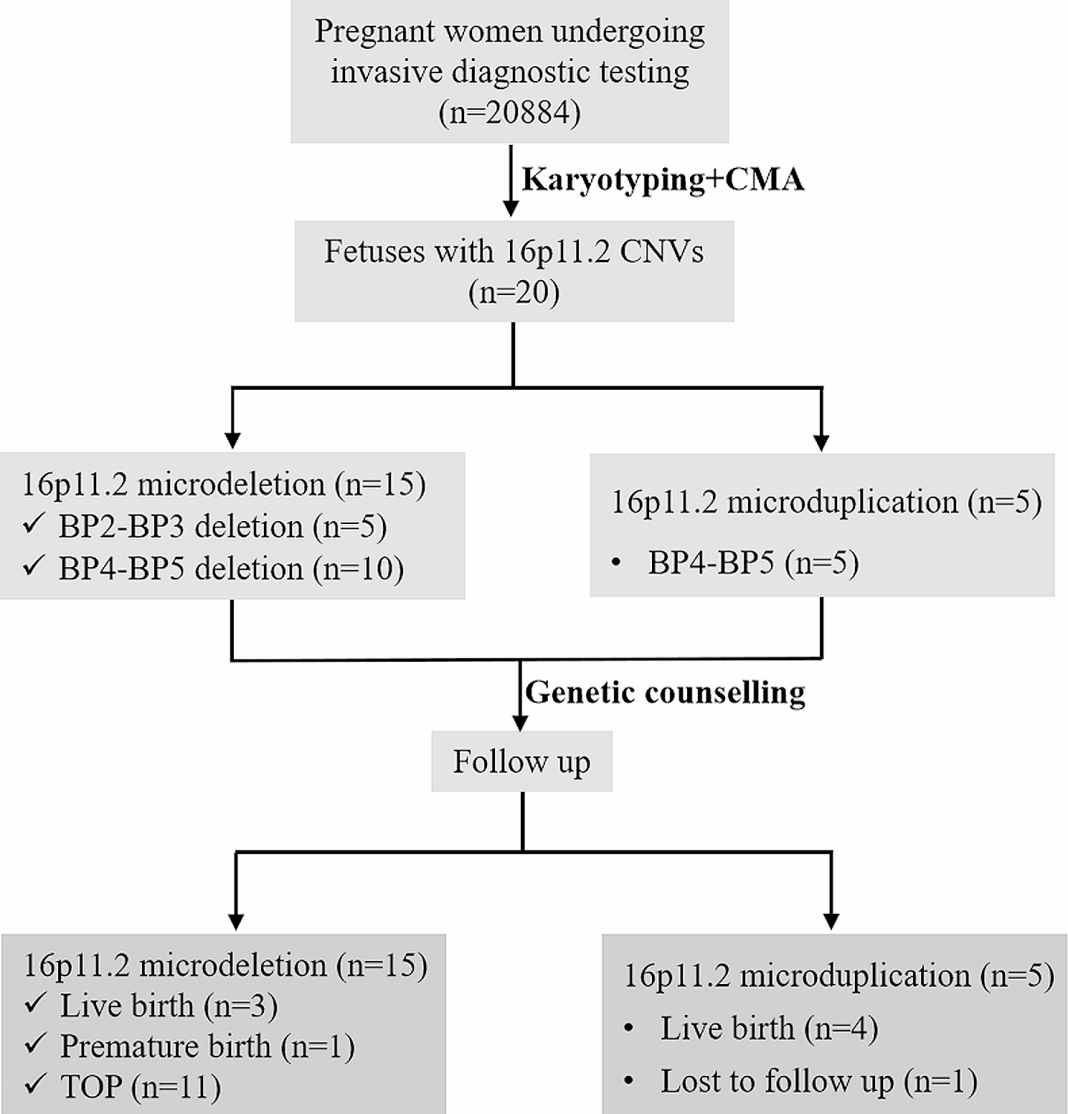
The flow chart of the study. CMA: chromosome microarray analysis; CNVs: copy number variations; TOP: termination of pregnancy

Of 20,884 pregnant women opting for prenatal invasive testing, 15 fetuses were identified with 16p11.2 microdeletions and five were diagnosed with 16p11.2 microduplications. The total detection rate of 16p11.2 CNVs was 0.10% (20/20,884) in prenatal setting. Detailed clinical data and follow-up of cases with 16p11.2 CNVs are shown in Fig. 1. Tables 1 and 2 summarize the clinical information for all the cases, mainly including gestational week, indications for prenatal diagnosis, parental phenotypes, CMA results, deleted/duplicated regions, inheritance, and pregnancy outcomes.

**Table 1:** Summary of clinical and molecular findings of fetuses presenting 16p11.2 microdeletions detected by CMA

Case No.	Age	Gravida and para	Gestational age (weeks)	Indications for prenatal diagnosis	Ultrasound findings during pregnancy	Parental phenotypes	Region	Breakpoint regions	Karyotype	CMA results (GRCh38)	Size (Mb)	Inheritance	Morbid genes	Pathogenicity	Pregnancy outcome		
															Gestational age	Length(cm)	Birth weight (kg)
1	31	G1P0	18+	NIPT infers high risk of chromosome 5	No evident anomalies	normal	16p11.2	BP2-BP3	46,XN	16p11.2(28,821,294–29,077,303)×1	0.256	*de novo*	TUFM, CD19, ATP21A, LAT	P	TOP at 25w		
2	32	G1P0	18+	NIPT infers high risk of chromosome 7	No evident anomalies	normal	16p11.2	BP2-BP3	46,XN	16p11.2(28,821,295–29,077,303)×1	0.256	pat	TUFM, CD19, ATP21A, LAT	P	TOP at 25w		
3	26	G2P0	16+	The mother’s karyotype:47,XXX	No evident anomalies	Mother: mild intellectual disability, non-Hodgkin’s lymphoma Father: deafness	16p11.2	BP2-BP3	46,XN	16p11.2(28,821,295–29,077,303)×1	0.256	n.a	TUFM, CD19, ATP21A, LAT	P	TOP at 22w		
4	21	G1P0	19+	Ultrasound abnormalities	Increased NT (9.9 mm)	normal	16p11.2 Xp22.33 or Yp11.32 Xp21.1	BP2-BP3	46,XN	16p11.2(28,737,295–29,039,870)×1 Xp22.33 or Yp11.32(990,171–1,449,101 or 940,171–1,399,101)×4 Xp21.1(31,710,803–31,944,832)×0	0.303 0.617 0.234	mat mat *de novo*	TUFM, CD19, ATP21A, LAT, DMD	P VOUS P	TOP at 28w		
5	40	G4P1	23+	AMA, ultrasound abnormalities, abnormal childbearing history: child with cerebral palsy	aberrant right subclavian artery, polyhydramnios	normal	16p11.2	BP2-BP3	46,XN	16p11.2(28,737,295–29,077,303)×1	0.34	*de novo*	TUFM, CD19, ATP21A, LAT	P	38w	58	3.1
6	28	G1P0	25+	Ultrasound abnormalities	VSD, pulmonic stenosis	normal	16p11.2	BP4-BP5	46,XN	16p11.2(29,597,003–30,178,708)x1	0.581	*de novo*	KIF22, PRRT2, TLCD38, ALDOA, TBX6	P	TOP		
7	20	G2P0A1	23+	Ultrasound abnormalities	Fetal growth restriction, short HL, polyhydramnios, nasal bone dysplasia	normal	16p11.2	BP4-BP5	46,XN	16p11.2(29,580,006–30,179,708)×1	0.599	mat	KIF22, PRRT2, TLCD38, ALDOA, TBX6	P	TOP at 31w		
8	32	G2P0A1	19+	Ultrasound abnormalities	Cleft lip and palate, echogenic bowel, polyhydramnios	normal	16p11.2	BP4-BP5	46,XN	16p11.2(29,580,006–30,178,708)×1	0.599	mat	KIF22, PRRT2, TLCD38, ALDOA, TBX6	P	Premature birth at 27w		
9	32	G3P0	24+	Ultrasound abnormalities	Renal agenesis, echogenic bowel	normal	16p11.2	BP4-BP5	46,XN	16p11.2(29,568,701–30,178,708)×1	0.61	*de novo*	KIF22, PRRT2, TLCD38, ALDOA, TBX6	P	TOP		
10	24	G1P0	20+	NIPT infers high risk of chromosome 16	No evident anomalies	Mother: mild learning disability, bradykinesia	16p11.2	BP4-BP5	46,XN	12p13.33(64,621–574,623)×1 16p11.2(29,555,975–30,178,708)×1	0.51 0.623	mat	KIF22, PRRT2, TLCD38, ALDOA, TBX6	VOUS P	39w	52	3900
11	25	G1P0	21+	Ultrasound abnormalities	Increased NT (2.83 mm)	normal	16p11.2	BP4-BP5	46,XN	16p11.2(29,417,211–30,178,708)×1	0.761	mat	KIF22, PRRT2, TLCD38, ALDOA, TBX6	P	Healthy		
12	27	G1P0	25+	Ultrasound abnormalities	Aberrant right subclavian artery	normal	16p11.2	BP4-BP5	46,XN	16p11.2(29,417,210–30,178,708)×1	0.761	n.a.	KIF22, PRRT2, TLCD38, ALDOA, TBX6	P	TOP at 29w		
13	30	G1P0	24+	Ultrasound abnormalities	Hemivertebra: the first lumbar vertebra is shaped like a “wedge”, and the thoracolumbar segment is slightly curved to the left side	Mother: right hydronephrosis	16p11.2	BP4-BP5	46,XN	16p11.2(29,417,210–30,178,708)×1	0.761	n.a.	KIF22, PRRT2, TLCD38, ALDOA, TBX6	P	TOP at 31w		
14	33	G2P1	18+	Abnormal childbearing history: an eight year-old cerebral palsy child who can not creep or sit before one year old died at the age of 11	No evident anomalies	Mother: speech difficulty Father: leukodermia	16p11.2	BP4-BP5	46,XN	16p11.2(29,329,271–30,178,708)×1	0.849	mat	KIF22, PRRT2, TLCD38, ALDOA, TBX6	P	TOP at 22w		
15	28	G3P0	24+	Ultrasound abnormalities	VSD, tricuspid atresia, right ventricular dysplasia, absence of nasal bone	normal	16p11.2	BP4-BP5	46,XN	16p11.2(29,417,210–30,333,637)×1	0.916	n.a.	KIF22, PRRT2, TLCD38, ALDOA, TBX6, CORO1A	P	TOP at 25w		

CMA: chromosomal microarray analysis, NIPT: non-invasive prenatal testing, P: pathogenic, TOP: termination of pregnancy, pat: paternal, n.a.: not available, NT: nuchal translucency, mat: maternal, VOUS: variants of unknown significance, AMA: advanced maternal age, VSD: ventricular septal defect, HL: humerus length

**Table 2:** Summary of clinical and molecular findings of fetuses presenting 16p11.2 microduplications detected by CMA

Case No.	Age	Gravida and para	Gestational age (weeks)	Indications for prenatal diagnosis	Ultrasound findings during pregnancy	Parental phenotypes	Region	Breakpoint regions	Karyotype	CMA results (GRCh38)	Size (Mb)	Inheritance	Morbid genes	Pathogenicity	Pregnancy outcome		
															Gestational age	Length(cm)	Birth weight (kg)
16	32	G1P0	19+	Voluntary request	No evident anomalies	normal	16p11.2	BP4-BP5	46,XN	16p11.2(29,617,340–30,178,708)×3	0.561	n.a.	KIF22, PRRT2, TLCD38, ALDOA, TBX6	P	Lost to follow up		
17	37	G1P0	18+	AMA, abnormal karyotype of the husband 46,XY, inv [2](p11.2q13)	No evident anomalies	normal	16p11.2	BP4-BP5	46,XN	16p11.2(29,580,006–30,165,919)×3	0.586	mat	KIF22, PRRT2, TLCD38, ALDOA, TBX6	P	Healthy		
18	36	G3P0	18+	AMA, recurrent miscarriage	No evident anomalies	Father: diabetes	16p11.2	BP4-BP5	46,XN	16p11.2(29,568,701–30,156,598)×3	0.588	pat	KIF22, PRRT2, TLCD38, ALDOA, TBX6	P	Healthy		
19	37	G3P1	19+	AMA	No evident anomalies	normal	16p11.2	BP4-BP5	46,XN	16p11.2(29,568,701–30,165,187)×3	0.596	*de novo*	KIF22, PRRT2, TLCD38, ALDOA, TBX6	P	38w	51	3.7
20	27	G2P0	18+	Risk of fetal trisomy 21: 1/15	No evident anomalies	normal	16p11.2	BP4-BP5	46,XN	16p11.2(29,401,182–30,178,708)×3	0.778	mat	KIF22, PRRT2, TLCD38, ALDOA, TBX6	P	39w	49	2.95

CMA: chromosomal microarray analysis, n.a.: not available, P: pathogenic, AMA: advanced maternal age, mat: maternal, pat: paternal

### Chromosomal anomalies detected by karyotyping

Amniotic fluid cells from all pregnant women were subjected to conventional karyotyping to determine whether there were balanced chromosomal rearrangements or mosaicism undetectable by CMA. Among the 15 identified 16p11.2 microdeletions and five 16p11.2 microduplications, no karyotypic anomalies were detected.

### Chromosome 16p11.2 microdeletions in affected fetuses

**Fig. 2:**
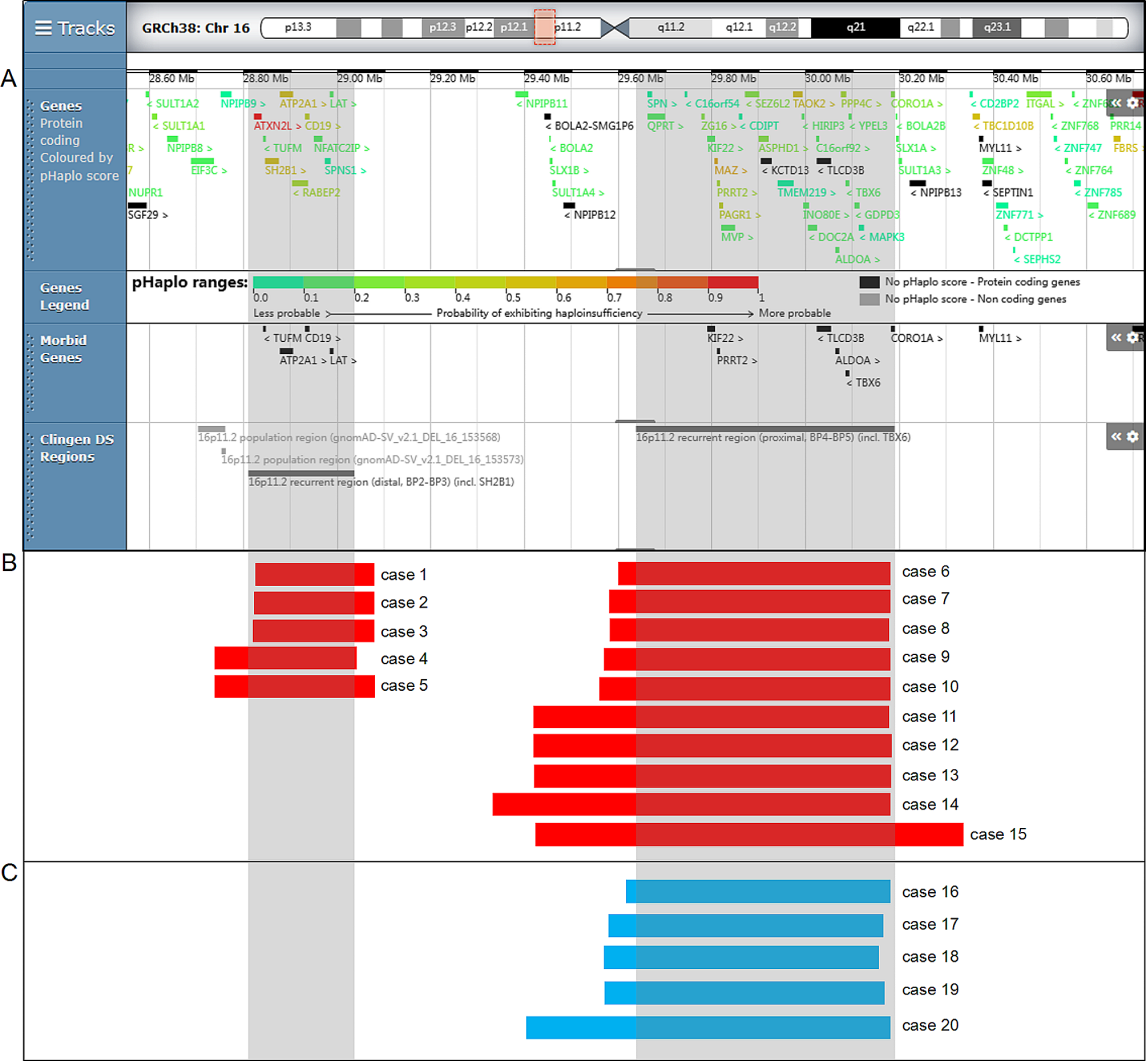
Scale representation of the deleted/duplicated region in the 16p11.2 region (https://decipher.sanger.ac.uk/): (**A**) Location of genes and genomic syndromes in the 16p11.2 locus; (**B**) Deleted fragments in the present cases; (C) Duplicated fragments in the present cases. Genomic parameters are from GRCh38/hg38

In our report, 15 cases (0.07%, 15/20,884) of 16p11.2 microdeletions were identified by CMA, ranging from 0.256 Mb to 0.916 Mb (Fig. 2B). 5/15 cases had 16p11.2 BP2-BP3 deletion and the overlapping region included four morbid genes (*TUFM*, *CD19*, *ATP2A1* and *LAT*). 10/15 cases had 16p11.2 BP4-BP5 deletion, with overlapping region covering five morbid genes (*KIF22*, *PRRT2*, *TLCD3B*, *ALDOA* and *TBX6*) (Fig. 2A). In addition, CMA detected a 0.234 Mb deletion of Xp21.1 and a 0.617 Mb duplication of Xp22.33 or Yp11.32 in case 4, and a 0.51 Mb deletion of 12p13.33 in case 10, the clinic pathogenicity of which was P, VOUS and VOUS, respectively. The distributions of indications for prenatal diagnosis were as follows: ultrasound anomalies (10/15), NIPT inferring chromosomal aneuploidy (3/15), abnormal childbearing history (2/15), advanced maternal age (1/15), and maternal abnormal karyotype (1/15). The abnormal ultrasound findings was recorded in 10/15 participants with 16p11.2 deletions, and the summarized frequency was as follows: polyhydramnios (3/10), cardiovascular malformations (2/10), echogenic bowel (2/10), aberrant right subclavian artery (2/10), increased NT (2/10), nasal bone absence or hypoplasia (2/10), hemivertebra (1/10), cleft lip and palate (1/10), renal agenesis (1/10), fetal growth restriction (FGR) (1/10), and short humerus length (1/10). Regarding the origins of the CNVs, 4/15 cases were *de novo*, 4/15 cases were unavailable. Parental inheritance was observed in 7/15 cases: the mother of case 10 presented mild learning disability and bradykinesia, and the mother of case 14 exhibited speech difficulty.

### Chromosome 16p11.2 microduplications in affected fetuses

A total of five fetuses (0.03%, 5/20,884) with 16p11.2 BP4-BP5 microduplications were detected in our study, ranging from 0.561 Mb to 0.778 Mb (Fig. 2C). The overlapping region covered five morbid genes, including *KIF22*, *PRRT2*, *TLCD3B*, *ALDOA* and *TBX6* (Fig. 2A). The indications for prenatal diagnosis were distributed as follows: advanced maternal age (3/5), risk of fetal trisomy 21 (1/5), recurrent miscarriage (1/5), voluntary request (1/5), and paternal chromosome anomaly (1/5). No ultrasound abnormalities were observed in any case during the pregnancy period. Among them, 3/5 cases were parentally inherited, 1/5 cases were *de novo*, and the origin of 1/5 cases was unavailable.

### Prenatal and postnatal follow-up assessment

Of the 15 16p11.2 deletions cases, 11 chose to terminate their pregnancies: three (case 1, 6 and 9) were *de novo*, four (case 2, 4, 7 and 14) were parental inheritance, and four (cases 3, 12, 13 and 15) were unavailable. Among the four cases opting for on-going pregnancy, case 5 carried a *de novo* 16p11.2 BP2-BP3 deletion, and the other three (cases 8, 10 and 11) carried maternally inherited 16p11.2 BP4-BP5 deletion. It was noteworthy that the mother of case 10 who presented mild learning disability and bradykinesia continued the pregnancy, and delivered a child with no visible abnormalities at birth. However, given the neonate’s young age, regular monitoring is necessary to detect any emerging abnormal symptoms. Among the five cases with 16p11.2 BP4-BP5 duplication, four (cases 17–20) chose to continue the pregnancies and gave birth to newborns with no visible abnormalities at birth while one (case 16) was lost to follow up.

We conducted follow-up on all neonates with 16p11.2 microdeletions and microduplications after birth, including congenital defects, craniofacial dysmorphisms, skeletal anomalies, as well as other developmental details. Overall, no visible abnormalities have been observed for these cases until this writing. Given the young age of all subjects, some abnormal clinical phenotypes may appear with increasing age. Neurological development assessment should be conducted, and long-term follow-up should be ensured until adulthood, with a particular focus on neurodevelopmental and behavioral disorders.

### Polled analysis of prenatally detected 16p11.2 CNVs

In Table 3, a total of 157 prenatal cases with 16p11.2 microdeletions were integrated from our study and the published literature [3, 10, 12–35]. Among these cases, 11.5% (18/157) were 16p11.2 BP2-BP3 deletions, and 88.5% (139/157) were 16p11.2 BP4-BP5 deletions. Abnormal ultrasound findings were observed in 70.1% (110/157) of these fetuses, of which 14 carried BP2-BP3 and 96 carried BP4-BP5 deletions, respectively. For 16p11.2 BP2-BP3 deletion, the summarized frequencies of recurrent abnormal ultrasound findings were as follows: FGR (3/18), single umbilical artery (3/18), aberrant subclavian artery (2/18), thickened nuchal translucency (NT) (2/18), and nasal bone absence or hypoplasia (2/18). A total of 61.1% (11/18) cases opted for termination of pregnancy (TOP) finally. For 16p11.2 BP4-BP5 deletion, the summarized frequencies of recurrent abnormal ultrasound findings were as follows: abnormality of the vertebral column or rib (33/139), thickened NT (14/139), renal anomalies (10/139), nasal bone absence or hypoplasia (10/139), ventricular/atrial septal defect (8/139), fetal ventriculomegaly (8/139), echogenic intracardiac foci (7/139), pulmonic stenosis (7/139), persistent left superior vena cava (5/139), aberrant subclavian artery (5/139), FGR (4/139), endocardial cushion defect (3/139), single umbilical artery (3/139), renal pyelectasis (3/139), polyhydramnios (3/139), limb anomaly (2/139), talipes equinovarus (2/139), supravalvular aortic stenosis/coarctation (2/139), orofacial region (2/139), echogenic bowel (2/139), and shortened femurs and humerus (2/139). 61.9% (86/139) cases of 16p11.2 BP4-BP5 deletion ultimately chose to terminate the pregnancies.

**Table 3:** The pooled data from all fetuses presenting 16p11.2 microdeletion

Characteristics	BP2-BP3		Total number	BP4-BP5		Total number
	Previous reports	Our study		Previous reports	Our study	
**Total number**	13	5	18	129	10	139
**Number of abnormal prenatal findings**	12	2	14	88	8	96
**Ultrasound findings**						
**Skeletal system**						
Abnormality of the vertebral column or rib	1		1	32	1	33
Talipes equinovarus				2		2
Limb anomaly	1		1	2		2
**Renal anomalies**				9	1	10
**Cardiovascular system**						
Ventricular/atrial septal defect				6	2	8
Pulmonic stenosis				6	1	7
Persistent left superior vena cava				5		5
Aberrant subclavian artery	1	1	2	4	1	5
Endocardial cushion defect				3		3
Supravalvular aortic stenosis/ coarctation	1		1	2		2
Right aortic arch	1		1	1		1
Endocardial fibroelastosis				1		1
Aortic straddle				1		1
Persistent right umbilical vein				1		1
Crossed pulmonary artery	1		1			
Tricuspid atresia					1	1
Right ventricular dysplasia					1	1
**Orofacial region**	1		1	1	1	2
**Brain anomalies**	1		1	1		1
**Chest anomalies**						
Congenital diaphragmatic hernia	1		1	1		1
Cystic adenomatiod malformation	1		1			
**Other anomalies**						
Thickened nuchal translucency	1	1	2	13	1	14
Nasal bone absence or hypoplasia	2		2	8	2	10
Fetal ventriculomegaly	1		1	8		8
Echogenic intracardiac foci	1		1	7		7
Fetal growth restriction	3		3	3	1	4
Single umbilical artery	3		3	3		3
Renal pyelectasis				3		3
Polyhydramnios		1	1	1	2	3
Echogenic bowel	1		1		2	2
Shortened femurs and humerus				1	1	2
Choroid plexus cysts				1		1
Thickened nuchal fold				1		1
**Termination of pregnancy**	7	4	11	79	7	86

In Table 4, a total of 23 prenatal cases with 16p11.2 microduplications were pooled from our study and the published literature [3, 24, 30, 33, 36–39]. Among these cases, 17.4% (4/23) were 16p11.2 BP2-BP3 duplications, and 82.6% (19/23) were 16p11.2 BP4-BP5 duplications. Abnormal ultrasound findings were observed in 34.8% (8/23) of these fetuses, including one case of BP2-BP3 duplication and seven cases of BP4-BP5 duplication. For 16p11.2 BP2-BP3 duplication, one case (1/4) presenting cardiac anomalies were observed. A total of 25% (1/4) cases opted for TOP. For 16p11.2 BP4-BP5 duplication, the summarized frequencies of abnormal ultrasound findings were as follows: polyhydramnios (2/19), right aortic arch (2/19), FGR (1/19), cardiac anomalies (1/19), increased nuchal fold (1/19), abdominal cystic masses (1/19), echogenic bowel (1/19), and absent nasal bone (1/19). A total of 21.1% (4/19) of these pregnancies were terminated.

**Table 4:** The pooled data from all fetuses presenting 16p11.2 microduplication

Characteristics	BP2-BP3		Total number	BP4-BP5		Total number
	Previous reports	Our study		Previous reports	Our study	
**Total number**	4		4	14	5	19
**Number of abnormal prenatal findings**	1		4	7		7
**Ultrasound findings**						
Polyhydramnios				2		2
Right aortic arch				2		2
Fetal growth restriction				1		1
Cardiac anomalies	1		1	1		1
Increased nuchal fold				1		1
Absent nasal bone				1		1
Abdominal cystic masses				1		1
Echogenic bowel				1		1
**Termination of pregnancy**	1		1	4		4

## Discussion

In our study, we retrospectively described 20 prenatal cases referred for prenatal invasive testing who were found to carry recurrent chromosomal 16p11.2 CNVs. The total detection rate of 16p11.2 CNVs was 0.10% in prenatal setting. Of all enrolled fetuses, five carried 16p11.2 BP2-BP3 deletion; 10 carried 16p11.2 BP4-BP5 deletion; and five carried 16p11.2 BP4-BP5 duplication. Various degrees of intrauterine phenotypic features, ranging from normal to abnormal, were noted in the cases with 16p11.2 deletions. No ultrasound anomalies were observed in cases with 16p11.2 duplications. Among the 15 cases of 16p11.2 microdeletions, 11 eventually chose TOP. For the five cases with 16p11.2 microduplications, four chose to continue their pregnancies and gave birth to healthy babies. To our knowledge, this is the largest cohort study with detailed prenatal phenotypes and follow-up for prenatally detected 16p11.2 CNVs in northeast China.

The incidence of 16p11.2 CNVs varies in different populations. In the general population, the prevalence of 16p11.2 deletions and duplications is 0.028–0.043% and 0.025–0.08%, respectively. For the individuals with neurodevelopmental disorders, the prevalence of 16p11.2 deletions and duplications increases to 0.25–2.9% and 0.15–0.78%, respectively [37, 40, 41]. In addition, ASD is found in approximately 25% of individuals with 16p11.2 CNVs and about 1% ASD patients carry 16p11.2 CNVs [42]. The prevalence of 16p11.2 CNVs in prenatal settings is sparsely described. In our study, the prevalence of 16p11.2 CNVs was 0.10% in prenatal series, with detection rates of 16p11.2 deletions and duplications being 0.07% and 0.03%, respectively. Although rarely reported, several studies have also reported the detection rate of 16p11.2 CNVs in the prenatal setting. According to the study of Lin et al. [12], the detection rate of 16p11.2 microdeletions in fetuses with abnormal ultrasound findings was approximately 0.5% (12/2262). Liu et al. [3] described 24 fetuses (24/8578, 0.28%) with 16p11.2 deletions and 6 fetuses (6/8578, 0.07%) with 16p11.2 duplications, with a total detection rate of 0.35%. Liu et al. [13] discovered that the prevalence of 16p11.2 deletions was 0.063% (55/86,035) in the prenatal period, which was similar to our study. In the study of Wang et al. [43], 1.63% of fetuses (81/4968) were diagnosed with 16p11.2 microdeletions, which was higher than other studies. Based upon the results mentioned above, the prevalence of 16p11.2 deletions and 16p11.2 duplications in prenatal period was 0.063–1.63% and 0.03–0.07%, respectively. More large-scale studies are needed to further clarify the frequencies of 16p11.2 CNVs in fetuses.

As one of the most frequent recurrent CNVs associated with neurodevelopmental disorders, the clinical features of 16p11.2 CNVs is characterized by phenotypic diversity and incomplete penetrance. Patients carrying 16p11.2 CNVs may exhibit a wide spectrum of clinic manifestations, including ID, ASD, ADHD, epilepsy, language disorders, schizophrenia, obesity, congenital malformations, and cardiovascular anomalies [33, 44–46]. So far, most of the published work involving 16p11.2 microdeletions and microduplications has focused on postnatal individuals, whereas the prenatal phenotypes in 16p11.2 CNVs are not well defined for the lacking of enough evidence, posing a challenge for prenatal genetic counseling for such cases.

Hence, to provide a better understanding of 16p11.2 CNVs in prenatal period, we made a pooled analysis of the fetuses carrying 16p11.2 microdeletions and microduplications based on the literature review (Tables 3 and 4). The specific breakpoints were classified into BP2-BP3 and BP4-BP5. The most common ultrasound findings in cases of 16p11.2 BP2-BP3 deletion included FGR and single umbilical artery. For 16p11.2 BP4-BP5 deletion, the three top structural malformations were abnormality of the vertebral column or rib, renal anomalies and ventricular/atrial septal defect; the three top non-structural malformations were thickened NT, nasal bone absence or hypoplasia, and fetal ventriculomegaly. It is noteworthy that echogenic bowel, observed in our cases, has not been reported in previous fetuses with 16p11.2 BP4-BP5 deletion. For 16p11.2 BP4-BP5 duplication, only polyhydramnios and right aortic arch were recurrent prenatal phenotypes. No recurrent prenatal phenotype was observed in 16p11.2 BP2-BP3 duplication. No ultrasound anomalies were observed in our cases of 16p11.2 BP4-BP5 duplication. Generally speaking, CNVs at the 16p11.2 locus can lead to a range of prenatal symptoms, from normal to abnormal, whether they are microduplications or microdeletions. 16p11.2 BP4-BP5 deletion could present some typical characteristics during the pregnancy period. For 16p11.2 BP2-BP3 deletion and 16p11.2 duplications, more clinical cases need to be accumulated to clarify the prenatal features. Refining the prenatal phenotypes associated with different breakpoints of 16p11.2 CNVs enables us to provide more accurate genetic counseling. In addition, we found that the incidence of ultrasound abnormalities in 16p11.2 deletions was higher than that in 16p11.2 duplications (*P* < 0.01). The rate of TOP in 16p11.2 deletions was also higher than that in 16p11.2 duplications (*P* < 0.01). The final pregnancy outcomes would probably be affected by multiple factors, including CNVs classification, the severity of ultrasound abnormalities, and possible future prognosis.

In our study, chromosome 16p11.2 BP4-BP5 CNVs were detected in 15 cases, including 10 16p11.2 deletions (cases 6–15) and five 16p11.2 duplications (cases 16 to 20). According to the DECIPHER database, 23 OMIM genes were located in the overlapping region, among which five were morbid genes associated with diseases (Fig. 2A). *TBX6* gene encodes a transcription factor, which is implicated in paraxial mesoderm development and somitogenesis during embryonic development. The haploinsufficiency of *TBX6* is supposed to play a critical role in the abnormal phenotypes of the skeleton and kidney. According to the OMIM database, the heterozygous or compound heterozygous mutations in the *TBX6* gene would cause spondylocostal dysostosis (SCDO5), characterized by developmental vertebral and rib defects [47]. Hemivertebra was observed in our case 13, which might be due to the haploinsufficiency of *TBX6*. In addition, heterozygous mutations of *TBX6* probably lead to genitourinary tract malformations, which might explain the renal agenesis observed in our case 9. It was reported that the increased *TBX6* gene dosages could also induce congenital cervical vertebral malformations in humans and mice, but these findings have not been reported in published prenatal cases till now [48]. *SEZ6L2* encodes a seizure-associated protein localized on the cell surface. It is regarded as a seizure-related gene [46]. The haploinsufficiency of *SEZ6L2* gene might also be associated with language delay, cognitive impairment, and autism [49]. Heterozygous mutations in the *KIF22* gene could cause spondyloepimetaphyseal dysplasia with joint laxity (SEMDJL), which is an autosomal-recessive skeletal dysplasia characterized by short stature, generalized joint laxity, slender hands, limb malalignment, and spinal deformity [50, 51]. *ALDOA* gene encodes fructose-1,6-bisphosphate aldolase A, and its mutations would cause Glycogen storage disease XII. Altering the *ALDOA* dosage will perturb energy metabolism at many stages in the brain and affect its development [52]. For *PRRT2* gene, there is sufficient evidence for haploinsufficiency (HI score:3) recorded in ClinGen database. The *PRRT2* mutations would lead to paroxysmal kinesigenic dyskinesia (PKD) and paroxysmal hypnogenic dyskinesia (PHD) in adults and self-limited familial neonatal-infantile epilepsy or infantile convulsion and choreoathetosis (ICCA) in infants [53]. The *TLCD3B* gene encodes the most highly expressed ceramide synthase in human retina. Its homozygous mutations would result in cone-rod dystrophy-22 (CORD22), which would lead to the loss of central vision due to the cone photoreceptor degeneration [54]. Some clinical evidence of other genes located in this region were described in other research. The haploinsufficiency of the *HIRIP3* gene was probably associated with cardiac arterial valve malformations [55]. *MAPK3* could regulate the neurodevelopment in ASD and schizophrenia [13]. With current knowledge, some evidence shows that some OMIM genes might be responsible for the abnormal phenotypes in the prenatal setting. Further studies are still needed to improve the understanding of the functions of the genes in this region.

Chromosome 16p11.2 BP2-BP3 deletions were identified in five cases (cases 1–5). According to the DECIPHER database, nine OMIM genes were located in the overlapping region, four of which are morbid genes associated with diseases (Fig. 2A). The *ATP2A1* gene encodes the fast-twitch skeletal muscle sarcoplasmic reticulum Ca(2+) ATPase; homozygous or compound heterozygous mutations in this gene cause Brody myopathy, characterized by exercise-induced impairment of muscle relaxation and stiffness [56]. The haploinsufficiency of *ATP2A1* may be associated with diaphragm malformations [17]. *ATP2A1* is also related to cardiac abnormalities [10]. Homozygous or compound heterozygous mutations in the *TUFM* gene cause combined oxidative phosphorylation deficiency 4, characterized by severe early-onset lactic acidosis and progressive fatal infantile encephalopathy [57]. The *CD19* and *LAT* genes are associated with immunodeficiency [10]. In addition, the disease-causing gene *SH2B1* encodes the Src homology 2B adaptor protein 1, which is involved in leptin and insulin signalling. There is little evidence for haploinsufficiency (HI score:1) recorded in ClinGen database for *SH2B1* gene, including developmental delay, severe obesity, hyperphagia and insulin resistance [9, 58]. Since the prenatal phenotypes of 16p11.2 BP2-BP3 CNVs were limited and untypical, additional clinical reports should be provided to further clarify the prenatal genotype-phenotype correlation.

Our study has some limitations. First, the subjects were collected in one single center, and the sample size is relatively small. Multi-center collaboration should be adopted to enlarge the sample size to establish a clearer correlation between 16p11.2 deletions/duplications and prenatal phenotypes in the future. Second, not all fetuses carrying 16p11.2 CNVs would exhibit abnormal ultrasound findings during the pregnancy period. Long-term follow-up, including postnatal evaluation, should be carried out regularly for those fetuses after birth. In addition, some single gene mutations detected using whole exome sequencing might also be the genetic etiology of the ultrasound anomalies, not just the pathogenic CNVs. Considering the incomplete penetrance and variable expressivity of 16p11.2 CNVs, further investigation is needed to establish a more detailed prenatal phenotype-genotype correlation.

## Conclusion

In this study, we delineated the clinical data and molecular findings in 20 prenatal cases carrying 16p11.2 deletions/duplications. For the first time, we summarized the prenatal features of 16p11.2 CNVs in diverse breakpoints based upon the published literature. 16p11.2 CNVs can manifest diverse prenatal phenotypes, ranging from normal to abnormal. For 16p11.2 BP4-BP5 deletion, the abnormality of the vertebral column or rib and thickened NT were the most common structural and non-structural abnormalities, respectively. In addition, echogenic bowel observed in our study might also be correlated with 16p11.2 BP4-BP5 deletion. 16p11.2 BP2-BP3 deletion was closely correlated with FGR and single umbilical artery. For 16p11.2 duplication, more clinic reports should be accumulated to clarify the prenatal manifestations. Since some abnormal phenotypes associated with 16p11.2 CNVs may not be recognizable in neonates, long-term follow-up is necessary regardless of whether they exhibit abnormal intrauterine phenotypic features or not.

## Data Availability

The datasets generated and/or analysed during the current study are available in the Gene Expression Omnibus repository, accession number GSE265911, https://www.ncbi.nlm.nih.gov/geo/query/acc.cgi?acc=GSE265911.

## Abbreviations

ADHD: attention deficit hyperactivity disorder
ASD: autism spectrum disorder
BD: bipolar disorder
CORD22: cone-rod dystrophy-22
CNVs: copy number variations
DD: developmental delay
DECIPHER: Database of Chromosomal Imbalance and Phenotype in Humans using Ensemble Resources
DGV: Database of Genomic Variants
ICCA: infantile convulsion and choreoathetosis
ID: intellectual disability
NAHR: non-allelic homologous recombination NIPTnon-invasive prenatal testing
NT: nuchal translucency
OMIM: Online Mendelian Inheritance in Man
PHD: paroxysmal hypnogenic dyskinesia
PKD: paroxysmal kinesigenic dyskinesia
SEMDJL: spondyloepimetaphyseal dysplasia with joint laxity
TOP: termination of pregnancy
